# Supplementary material for: Socioeconomic inequalities, substance use, and chronic multimorbidity in Kiambu and Nakuru counties in Kenya
Source: PLOS Glob Public Health. 2025 Dec 8;5(12):e0005544. doi: 10.1371/journal.pgph.0005544 (PMC12685187; doi:10.1371/journal.pgph.0005544)

PLOS Global Public Health

Multimorbidity of Chronic Diseases and Association with Substance Use in Kiambu and Nakuru Counties in Kenya

--Manuscript Draft--

**Manuscript Number:**	PGPH-D-25-01204
**Article Type:**	Research Article
**Full Title:**	Multimorbidity of Chronic Diseases and Association with Substance Use in Kiambu and Nakuru Counties in Kenya
**Short Title:**	Chronic Disease Multimorbidity and Substance Use in Kenya
**Corresponding Author:**	Linnet Ongeri, M.D, PhD<br>Kenya Medical Research Institute<br>Nairobi, KENYA
**Order of Authors:**	Linnet Ongeri, M.D, PhD
	Lydia Kaduka, PhD
	Damaris Matoke, PhD
	Doris Njomo, PhD
	Zipporah Bukania, PhD
	Moses Mwangi
	Linus Ndegwa
	Joanna Olale
	Caleb Othieno, MD,PhD
	Sahara Hussein
	Geoffrey Barini
	Valentine Wanga, PhD
	Polycarp Mogeni, PhD
**Keywords:**	Multimorbidity<br>Non Communicable Diseases<br>Substance use<br>Alcohol use<br>Depression<br>Hypertension<br>Diabetes<br>Obesity<br>household survey<br>Kenya<br>chronic diseases
**Abstract:**	Multimorbidity is an emerging public health concern due to its significant individual and population-level impacts, including increased healthcare costs and reduced quality of life. Alcohol and substance use play a complex role in the development and exacerbation of multimorbid conditions. However, the interplay between substance use and multimorbidity remains underexplored, particularly in sub-Saharan Africa. This study examined the prevalence of multimorbidity and associated factors within a sub-Saharan African context. We employed a stratified, two-stage random sampling design for a household-based cross-sectional survey in two counties in central Kenya. Participants were assessed using standardized questionnaires for chronic conditions. Data were collected via interviews and entered electronically using the ODK platform. Tetrachoric correlation was used for pairwise correlations between chronic conditions, whilst the weighted modified Poisson regression model, accounting for the complex survey design, was used to examine associations between multimorbidity and sociodemographic factors. We recruited 1,484 participants with a median age of 36

Powered by Editorial Manager® and ProduXion Manager® from Aries Systems Corporation

	years. Overall, 58% were female, 60% were married, and over half (52%) reported a lifetime history of substance use. Multimorbidity was prevalent in 7.7% of participants and was significantly associated with older age (adjusted prevalence ratio (aPR), 12.52 [95% CI, 3.68–42.56]; P<0.001), female gender (aPR, 1.62 [95% CI, 1.16–2.27]; P=0.005), being divorced or separated (aPR, 2.22 [95% CI, 1.37–3.62]; P=0.002), current alcohol use (aPR, 1.68 [95% CI, 1.07–2.64]; P=0.024) and lifetime alcohol use (aPR, 1.65 [95% CI, 1.04-2.62]; P=0.034). In contrast, a secondary level education was protective against multimorbidity (aPR, 0.58 [95% CI, 0.35–0.96]; P=0.035). These findings underscore the need for integrated, community-based approaches to prevent and manage multimorbidity, particularly among older adults, women, and individuals with a history of alcohol or substance use.
**Additional Information:**	
**Question**	**Response**
**Financial Disclosure**<br><br>Enter a financial disclosure statement that describes the sources of funding for the work included in this submission and the role the funder(s) played. This includes grants and any commercial funding of the work or authors.<br><br>This statement will be typeset if the manuscript is accepted for publication.<br><br>*Please review the submission guidelines and the instructions link below for detailed requirements and guidance.*	This study was funded by the Kenya Medical research Internal Research Grants (grant number L 0178). L.O received the funding award. The funders had no role in study design, data collection and analysis, decision to publish or preparation of manuscript.
**Competing Interests**<br><br>On behalf of all authors, disclose any competing interests that could be perceived to bias this work.<br><br>This statement will be typeset if the manuscript is accepted for publication.<br><br>*Please review the instructions link below and PLOS Global Public Health's competing interests policy to determine what information must be disclosed at submission.*	The other authors declare no conflict of interest.
**Data Availability**	Data used in this analysis will be published online once all protocol objectives are completed. Reasonable requests for data can be directed to the corresponding author.

Powered by Editorial Manager® and ProduXion Manager® from Aries Systems Corporation

Before publication, Authors are required to make fully available and without restriction all data underlying their findings. Please see our PLOS Data Policy page for detailed information on this policy.<br><br>A **Data Availability Statement**, detailing where the data can be accessed, is required at first submission. Insert your Data Availability Statement in the box below.<br><br>Please see the data reporting section of our submission guidelines for instructions on what you need to include in your Data Availability Statement.<br><br>This statement will be typeset if the manuscript is accepted for publication.<br><br>*PLOS allows rare exemptions to address legal and ethical concerns. If you have legal or ethical restrictions, please use the box below to detail these in full sentences for the Journal team to consider.*	

Manuscript

Click here to access/download;Manuscript;Revised Multimorbidity and SU Manuscript-v2_20_05_2025.pdf

Multimorbidity of Chronic Diseases and Association with Substance Use in Kiambu and Nakuru Counties in Kenya

Linnet Ongeri[1], Lydia Kaduka[2], Damaris Matoke[3], Doris Njomo[4], Zipporah Bukania[2], Moses Mwangi[2], Linus Ndegwa[1], Joanna Olale[1], Caleb Othieno[5], Sahara Hussein[1], Geoffrey Barini[6], Valentine Wanga[7], Polycarp Mogeni[1].

[1]Centre for Clinical Research, Kenya Medical Research Institute, Nairobi, Kenya

[2]Centre for Public Health Research, Kenya Medical Research Institute, Nairobi, Kenya

[3]Centre for Biotechnology Research and Development, Kenya Medical Research Institute, Nairobi, Kenya

[4]Eastern and Southern Africa Centre of International Parasite Control, Nairobi, Kenya

[5]Department of Medicine, Maseno University, Kisumu, Kenya

[6]Department of Pure and Applied Mathematics, Jomo Kenyatta University of Agriculture and Technology, Nairobi, Kenya.

[7]University of Washington, Seattle, WA, USA.

Corresponding author

Email: longeri@kemri.go.ke (L.O)

Abstract

Multimorbidity is an emerging public health concern due to its significant individual and population-level impacts, including increased healthcare costs and reduced quality of life. Alcohol and substance use play a complex role in the development and exacerbation of multimorbid conditions. However, the interplay between substance use and multimorbidity remains underexplored, particularly in sub-Saharan Africa. This study examined the prevalence of multimorbidity and associated factors within a sub-Saharan African context. We employed a stratified, two-stage random sampling design for a household-based cross-sectional survey in two counties in central Kenya. Participants were assessed using standardized questionnaires for chronic conditions. Data were collected via interviews and entered electronically using the ODK platform. Tetrachoric correlation was used for pairwise correlations between chronic conditions, whilst the weighted modified Poisson regression model, accounting for the complex survey design, was used to examine associations between multimorbidity and sociodemographic factors. We recruited 1,484 participants with a median age of 36 years. Overall, 58% were female, 60% were married, and over half (52%) reported a lifetime history of substance use. Multimorbidity was prevalent in 7.7% of participants and was significantly associated with older age (adjusted prevalence ratio (aPR), 12.52 [95% CI, 3.68–42.56]; P<0.001), female gender (aPR, 1.62 [95% CI, 1.16–2.27]; P=0.005), being divorced or separated (aPR, 2.22 [95% CI, 1.37–3.62]; P=0.002), current alcohol use (aPR, 1.68 [95% CI, 1.07–2.64]; P=0.024) and lifetime alcohol use (aPR, 1.65 [95% CI, 1.04-2.62]; P=0.034). In contrast, a secondary level education was protective against multimorbidity (aPR, 0.58 [95% CI, 0.35–0.96]; P=0.035). These findings underscore the need for integrated, community-based approaches to prevent and manage multimorbidity, particularly among older adults, women, and individuals with a history of alcohol or substance use.

Introduction

Multimorbidity, defined as the presence of two or more long-term health conditions in a person at the same time, differs from comorbidity, which refers to additional conditions experienced by a person who has a specific condition(1). Multimorbid conditions focus on a holistic approach to care and can include physical or mental health conditions, ongoing conditions, symptom complexes, sensory impairment and alcohol or substance misuse(2). The growing prevalence of multimorbidity globally has a significant impact on health care systems, particularly in low- and middle-income countries (LMICs)(2).

In Kenya, chronic diseases including, hypertension, diabetes and mental health conditions carry a significant public health challenge(3). These conditions account for a substantial proportion of morbidity and mortality contributing to over half of hospital admissions and a rising burden of disability(3). Specifically, over 30% of all deaths are attributable to Non Communicable Diseases (NCDs)(3). The dual burden of infectious and chronic diseases in Kenya worsens the strain on health care systems, systems that are often under-resourced and ill equipped to handle long term care needs(4). Affected populations face considerable challenges, including limited access to affordable healthcare, inadequate disease management support, and high out-of-pocket costs, which can lead to catastrophic financial consequences(5,6). Additionally, the prevalence of risk factors such as unhealthy diets, physical inactivity, tobacco use, and harmful alcohol consumption further increases the disease burden, particularly in urban and peri-urban settings(7–9). Addressing chronic diseases in Kenya is critical for improving population health outcomes and reducing the long-term economic impact of these conditions(3).

Substance use, including the use of alcohol, tobacco and other substances, is a well-established risk factor affecting both onset and exacerbation of chronic diseases(10). Excessive alcohol consumption has been linked to conditions such as liver disease, hypertension and cancer. It can also weaken the immune system thus increasing vulnerability to infection(11,12). Other substance use including stimulants and opioids are also associated with cardiovascular diseases and mental health disorders(13,14). Substance use complicates disease management and significantly increases the likelihood of multimorbidity through poor adherence to treatment, physiological stress, interaction with medications and delayed health seeking behaviour(15–17).

Furthermore, substance use disorders frequently co-occur with mental health disorders such as depression further complicating the management of chronic diseases(18,19).

The prevalence of diabetes in Kenya is estimated at approximately 3% of the adult population, with higher rates observed in urban settings (3.5%) compared to rural areas (2.7%) (20). Hypertension, on the other hand, affects about 25% of the adult population (21). Depression in the African region has a prevalence of 9% (22) but this varies in Kenya depending on assessment tools and population studied. Among people living with HIV, the prevalence of depression is 13.8%(23), while in patient populations, it is higher at 19% (24) compared to 12.3% in the general population(25). Regarding substance use, alcohol use disorders are the most prevalent globally, affecting over 100 million people. Harmful alcohol use contributes to approximately 3 million deaths annually, accounting for 5.1% of the global burden of disease and injury (26). In Kenya, the past month prevalence of substance misuse is 18% with alcohol (11.8%) and tobacco (8.5%) being the most commonly used substances(27). Substance use patterns vary significantly by type of substance and geographical region, even within the same country. This highlights the need for region- specific epidemiological surveys to better understand substance use patterns and the populations affected. Research in substance use epidemiology has demonstrated consistent links with several socio-demographic variables. Rates of these disorders are found usually to increase in community surveys as a function of male sex (28), younger age (29), lower education (30), non-partnered status(31), low income (32) and other variables indicative of social disadvantage that often concentrate together within population subgroups. Health system challenges in addressing both multimorbidity and substance use in resource constrained settings exist.

There is a paucity of data assessing the interplay between multimorbidity and substance use, particularly within the African context. Although few studies have explored the occurrence of chronic diseases individually, limited data exist on their concurrent impact and association with substance use. Understanding the burden of multimorbidity and its association with substance use in Kenya is crucial for informing tailored public health interventions and developing effective policies to address these critical public health challenges. This study aims to fill this critical knowledge gap by determining the prevalence of multimorbidity in a general population and assessing its association with substance use in two populous counties in Kenya.

Materials and Methods

Ethical statement

The study was conducted in two counties in Kenya with ethical approval from the KEMRI Scientific and Ethics Review Unit (KEMRI; SERU #3254) and ~~was conducted~~ in compliance with the World Medical Association Helsinki Declaration on the ethical conduct of research involving human subjects. All participants were informed about the purpose, risks, benefits, and study procedures, and written informed consent was obtained before data collection.

Study setting

This cross-sectional survey targeted adults residing in Kiambu and Nakuru Counties in Kenya (Fig 1). Kiambu County, located in Central Kenya, borders Nairobi County to the north, which hosts Kenya's capital city. It has a population of approximately 2,417,73 (33) and covers 2,543 square kilometers. The county's population is 60% urban and 40% rural, reflecting the influence of Nairobi's growth (33). Nakuru County, situated approximately 90 kilometers from Nairobi in the Rift Valley region, has a population of 2,162,202, making it the third most populous county after Nairobi and Kiambu (33). It spans 7,495.1 square kilometers and includes eleven constituencies and thirteen administrative towns (Fig 1). These two counties in central Kenya were selected for their balanced urban/rural population distribution and representation of Kenya's regional and ethnic diversity, with Kiswahili and Kikuyu being the predominant languages.

Figure 1: Map of Kenya showing the various county boundaries and the study areas. The Kenya administrative boundary Shape files were retrieved from (https://data.humdata.org/dataset/cod-ab-ken). Maps were generated using open-source QGIS version 3.34.2 (https://qgis.org/).

Study participants

This study involved adults aged 18 years or older who were residing in Kiambu and Nakuru counties in Kenya. A team of trained research assistants with clinical backgrounds recruited participants in their households. To be eligible, participants had to be residents of the selected households for at least six months, be available for the study visit, and understand English or Kiswahili, as the study instruments were translated into both languages.

Study design and sampling

The minimum sample size was calculated using the Fisher exact formula(34), assuming a 50% prevalence of drug and substance use, a 95% confidence interval (Z=1.96), and an absolute precision of 0.025. This resulted in an estimated sample size of 1537 participants. Accounting for a 10% non-response rate, the final sample size was adjusted to 1691 participants. Based on proportional population distribution from the 2009 census, 850 participants were allocated to Kiambu County (population 1,623,282) and 841 participants to Nakuru County (population 1,603,325). The sampling frame was based on the National Sample Survey and Evaluation Programme (NASSEP V) maintained by the Kenya National Bureau of Statistics (KNBS), utilizing 2009 census maps to construct a survey-specific sampling frame.

A two-stage, cluster sampling design was employed. In the first stage, a probability proportional to size (PPS) sampling method was used to select a random sample of Enumeration Areas (EAs) from a comprehensive list of all EAs within the target population stratified by county. EAs, characterized by 50 to 149 households with an average of 100 residents, were selected based on detailed maps outlining their boundaries, structures, and population distribution by gender. In the second stage, a simple random sample of households was drawn from each selected EA. Within each selected household, one eligible adult aged 18 years or older was randomly selected using the KISH Grid method (35) to ensure unbiased participant selection.

Data collection procedures

Data collection was conducted by trained research assistants between 2nd May 2017 and 15th July 2017. Efforts were made to interview participants in selected households, including follow-up visits to those initially unavailable to minimize non-response bias. If no household member was available during the additional visit, no replacement was made. For households with eligible members present, the KISH Grid method was employed to randomly select one participant. This involved documenting all eligible residents, recording their age and gender, and using a pre-assigned table of random numbers to select the respondent. This method ensured unbiased selection and minimized potential selection bias.

Data was collected using tablets pre-loaded with study questionnaires programmed within the Open Data Kit (ODK) system. To reduce data entry errors, the entry screens were pre-programmed to accept only codes within a predetermined range. Interviews were administered in English or Kiswahili, tailored to the participant's language of preference. Before initiating the interview, written informed consent was obtained from all study participants after they were explicitly informed of their right to withdraw participation at any given time, including the option to decline participation in any invasive procedures while still consenting to the interview.

Assessment of chronic conditions

The presence of diabetes and hypertension was determined through a combination of self-reported current treatment and objective measurements. Diabetes was diagnosed based on elevated blood glucose levels- random blood sugar ≥11mmol/L, fasting blood sugar ≥7mmol/L or self-reported use of diabetes medications. Hypertension was diagnosed based on elevated blood pressure readings (systolic pressure ≥140 mmHg or diastolic pressure ≥90 mmHg) or taking antihypertensive medications. Obesity was defined as a BMI greater than 30 kg/m2, while underweight was defined as a BMI less than 18.5 kg/m2. Nutritional assessments included anthropometric measurements, specifically weight and height, used to calculate the BMI. Clinical assessments encompassed blood pressure measurements using an Omron M6 device after a 30-minute rest period, during which participants were advised to abstain from smoking, coffee, or other stimulants. Three blood pressure readings were taken on the right arm, and the average was recorded. Random Blood Sugar (RBS) levels were assessed for diabetes assessments. Depressive symptoms were assessed using the 10-item Patient Health Questionnaire (PHQ-9), a widely recognized screening tool for depression (36). Respondents reported the frequency with which they had experienced the following symptoms two weeks preceding the survey: *'little interest or pleasure in doing things', 'feeling down, depressed or hopeless', 'trouble falling asleep, staying asleep, or sleeping too much', 'feeling tired or having little energy', 'poor appetite or overeating', 'feeling bad about yourself - or that you're a failure or have let yourself or your family down', 'trouble concentrating on things, such as reading the newspaper or watching television', 'moving or speaking so slowly that other people could have noticed or, the opposite - being so fidgety or restless that you have been moving around a lot more than usual, 'thoughts that you*

would be better off dead or of hurting yourself in some way'. Responses were scored on a four-point Likert scale, from 0 (never) to 3 (always), with the total score for all the questions (PHQ-9 score) ranging from 0 to 27. A PHQ-9 score ≥10 was considered clinically significant depressive symptoms (36). All procedures followed standardized protocols to ensure the accuracy and reliability of collected data.

Outcome

The main outcome of interest was multimorbidity, defined as the occurrence of two or more chronic conditions, including obesity, depression, underweight, diabetes, and hypertension.

Demographic and exposure variables

Demographic data collected included age, sex, level of education, employment status, income, and marital status. The questionnaire, translated into Kiswahili, was pre-tested and refined before training and fieldwork commenced. Patterns of substance use were assessed using the WHO Core Model Questionnaire, a standardized tool developed by the World Health Organization to gather comprehensive data on substance use behaviors. The questionnaire collects valuable insights into the types of substances used, frequency of use, and related behaviors (37). The key exposure variables included current or previous use of the following substances: alcohol, tobacco, cannabis, amphetamine and amphetamine type stimulants, sedatives and hypnotics, tranquillizers, hallucinogens, cocaine, opium, heroin, and volatile inhalants. We also assessed for derived exposures, including any substance use (defined as the use of at least one substance such as alcohol, tobacco, or any other WHO-defined substance) and use of multiple substances (polydrug: defined as the use of >2 substances).

Statistical analysis

Descriptive statistics, including means and standard deviations for continuous variables and proportions for categorical variables, were calculated, accounting for the complex survey design and using the Taylor linearization to estimate standard errors. The tetrachoric correlation was used to analyze pairwise correlations between chronic conditions and multimorbidity. To account for the complex survey design, a weighted modified Poisson regression model was used to assess the association between multimorbidity and socioeconomic and demographic factors (age, sex,

marital status, education, and income) using a backward stepwise procedure. Furthermore, we examined the associations between multimorbidity and each substance use variable, adjusting for the aforementioned socioeconomic and demographic factors. The variance inflation factors (VIF) were calculated to assess multicollinearity among the socioeconomic and demographic factors in each separate regression model, analyzing the association for each substance use variable. The various substance use combinations were defined a priori and were not adjusted for each other, given the expected correlation between different substances and combinations. The analyses presented herein are exploratory and did not incorporate a correction for multiple comparisons. We reported adjusted prevalence ratios (aPR) and 95% confidence intervals (95% CI). Statistical significance was set at $p < 0.05$. All analyses, except for the Tetrachoric correlations, accounted for the complex survey design and were conducted using Stata/SE 18 (38).

Results

Participant characteristics and substance use summaries

In total, 1484 study participants were recruited. The median age was 36 years (interquartile range IQR: 27 to 49), 58.3% (n=859) were female, 60.9% (n=904) were married, 15.5% (n=230) had college/university level education, and 15.0% (n=222) reported earning ≥Ksh.20,000. Overall, 52.6% (n=780) reported ever using at least one substance, and 29.1% (n=432) reported ever use of multiple substances. Among substance users, the most common substances used were alcohol (48.7%) and smoking cigarettes or tobacco (22.6%).

Prevalence of chronic conditions and multimorbidity

The prevalence of multimorbidity among the study population was 7.7% (n=115). The prevalence of multimorbidity by socioeconomic and demographic status, and substance use are described in detail in Table1 and Table 2. Among the individual chronic diseases examined, 7.0% (n=104) reported depressive symptoms, 2.2% (n=31) were categorized as having diabetes, 12.6% (n=183) had hypertension, 7.6% (n=108) were underweight, and 17.7% (n=250) were obese (Figure 2). The common co-occurrence combination was hypertension and obesity (n=42), followed by underweight and depression (n=14), obesity and depression (n=14), and hypertension and

depression (n=14). One individual had more than 3 of the 5 chronic conditions examined (Figure 2).

Figure 2: Frequency of unique combinations of chronic disease comorbidity. The displayed raw data has not been adjusted for the survey study design. The filled blue circles on the lines show the comorbidity of chronic diseases that are captured by the corresponding bar.

Table 1: Socioeconomic and demographic characteristics of study participants, showing multimorbidity prevalence (with 95% CI) and raw numbers for each factor.

	**Total (N)**	Multimorbidity **n(%)**	**(95%CI)**
**Overall**	1484	115 (7.7)	(6.4, 9.2)
**Age group**			
18 - 24	278	3 (1.1)	(0.2, 3.1)
25 - 35	488	21 (4.3)	(2.7, 6.5)
36 - 45	309	18 (5.8)	(3.5, 9.1)
46+	410	73 (17.8)	(14.2, 21.9)
**Sex**			
Male	615	36 (5.9)	(4.1, 8.0)
Female	859	79 (9.2)	(7.3, 11.3)
**Marital status**			
Married	904	67 (7.4)	(5.8, 9.3)
Separated/Divorced	103	22 (21.4)	(13.9, 30.5)
Single	366	9 (2.5)	(1.1, 4.6)
Widowed/Widower	112	18 (16.1)	(9.8, 24.2)
**Education level**			
Primary or less	719	81 (11.3)	(9.0, 13.8)
Secondary	535	24 (4.5)	(2.9, 6.6)
College/University	230	10 (4.3)	(2.1, 7.9)
**Income last month in KSh.**			
<1,000	220	24 (10.9)	(7.1, 15.8)
>=1,000 to <10,000	764	59 (7.7)	(5.9, 9.8)
>=10,000 to <20,000	278	18 (6.5)	(3.9, 10.0)
>20,000	222	14 (6.3)	(3.5, 10.4)

Table 2: Prevalence of multimorbidity (with 95% CI) by substance use categories among study.

	Total (N)	Co-morbidity n (%)	(95%CI)
**Overall**	1484	115 (7.7)	(6.4, 9.2)
**Currently on any substance**			
Yes	385	28 (7.3)	(4.9, 10.3)
No	1099	88 (8.0)	(6.5, 9.8)
**Ever used any substance**			
Yes	780	67 (8.6)	(6.7, 10.8)
No	704	48 (6.8)	(5.1, 8.9)
**Ever used multiple substances**			
Yes	432	28 (6.5)	(4.3, 9.2)
No	1052	87 (8.3)	(6.7, 10.1)
**Currently using multiple substances**			
Yes	168	9 (5.4)	(2.5, 9.9)
No	1316	106 (8.1)	(6.6, 9.7)
**Ever used multiple substances**			
<2	704	48 (6.8)	(5.1, 8.9)
2	348	39 (11.2)	(8.1, 15.0)
3 or more	432	28 (6.5)	(4.3, 9.2)
**Currently using multiple substances**			
<2	1099	88 (8.0)	(6.5, 9.8)
2	216	18 (8.3)	(5.0, 12.9)
3 or more	168	9 (5.4)	(2.5, 9.9)
**Currently smoke cigarettes**			
Yes	203	13 (6.4)	(3.5, 10.7)
No	1282	102 (8.0)	(6.5, 9.6)
**Currently smoke Cigarettes or tobacco**			
Yes	371	27 (7.3)	(4.9, 10.4)
No	1113	88 (7.9)	(6.4, 9.7)
**Ever taken alcohol**			
Yes	723	65 (9.0)	(7.0, 11.3)
No	761	50 (6.6)	(4.9, 8.6)
**Currently taking alcohol**			
Yes	270	23 (8.5)	(5.5, 12.5)
No	1214	92 (7.6)	(6.2, 9.2)

The prevalence of multimorbidity was higher in older age groups, rising from 1.1% (95% CI: 0.2 to 3.1) among adults aged 18-24 years to 17.8% (95% CI: 14.2 to 21.9) among those aged over 45 years (Table 1). No marked sex differences were observed in the overall prevalence of multimorbidity. An analysis of individual chronic diseases revealed a higher prevalence of obesity, hypertension, and diabetes among older age groups. Sex disparities were evident only among individuals with obesity, with women experiencing a higher prevalence compared to men (Figure 3).

Figure 3: Prevalence of multimorbidity and chronic conditions by age (adults ≥18 years) in two Kenyan counties, with 95% confidence intervals (binomial exact method).

Pairwise correlations between chronic illnesses were generally weak, except for a moderate correlation between diabetes and hypertension (Tetrachoric correlation [r] = 0.5). In contrast, stronger correlations were observed between multimorbidity and individual chronic illnesses, ranging from r = 0.63 to 0.82, except for underweight (r = 0.28). Table 3 provides further details on the observed associations between chronic conditions and substance use. Obesity had a moderate positive correlation with diabetes (r=0.32). Substance use variables were strongly intercorrelated (e.g., ever used multiple substances and current multiple substance use, r=1), indicating close associations between these behaviors. Interestingly, depressive symptoms were moderately associated with multimorbidity (r=0.71).

Table 3: Tetrachoric pairwise correlations between multimorbidity, chronic conditions, and substance use disorders among study participants. Blue and green represent significant positive and negative correlations, respectively.

	Multimorbidity	Depressive symptoms	Obesity	Underweight	Hypertension	Diabetes	Ever used any substance	Currently on any substance	Ever used multiple substances	Current multiple substance use	Current cigarette or tobacco smoker	Currently taking alcohol	Ever taken alcohol
Multimorbidity	1												
Depressive Symptoms	0.71	1											
Obesity	0.63	-0.05	1										
Underweight	0.28	0.16	-1	1									
Hypertension	0.82	0.15	0.24	-0.22	1								
Diabetes	0.78	0.25	0.32	-1	0.50	1							
Ever used any substance	0.08	0.18	-0.18	0.09	0.05	0.08	1						
Currently on any substance	-0.05	0.13	-0.22	0.23	-0.03	-0.18	1	1					
Ever used multiple substances	-0.09	0.21	-0.35	0.19	0.01	-0.21	1	0.86	1				
Current multiple substance use	-0.14	0.11	-0.33	0.24	-0.11	-0.11	1	1	1	1			
Current cigarette or tobacco smoker	-0.05	0.16	-0.40	0.21	0.05	-0.1	1	0.79	0.97	0.83	1		
Currently taking alcohol	0.02	0.15	-0.18	0.22	-0.01	-0.08	1	1	0.72	0.90	0.64	1	
Ever taken alcohol	0.09	0.20	-0.19	0.09	0.07	0.08	1	0.83	0.91	0.73	0.79	1	1

Risk factors of multimorbidity among study participants

Multimorbidity was higher among participants 46 years and older (aPR, 12.52 [95% CI, 3.68–42.56]; P<0.001), females (aPR, 1.62 [95% CI, 1.16–2.27]; P=0.005) and those who reported separation or divorce (aPR, 2.22 [95% CI, 1.37–3.62]; P=0.002). However, completing secondary education was associated with a decreased likelihood of multimorbidity (aPR, 0.58 [95% CI, 0.35–0.96]; P=0.035) (Table 4).

Table 4: Association between socio-economic and demographic factors, and multimorbidity among study participants. Model 1 presents results from the univariable modified Poisson regression model, and Model 2 presents results from a multivariable modified Poisson regression model.

	Model 1: Univariable Analysis			Model 2: Multivariable Analysis		
	PR	95% CI	p-value	PR	95% CI	p-value
Age group (ref. 18-24)						
25 - 35	4.77	(1.29,17.63)	0.020	3.35	(0.88,12.82)	0.076
36 - 45	6.29	(1.77,22.43)	0.005	4.32	(1.15,16.28)	0.031
46+	19.38	(6.07,61.87)	0.000	12.52	(3.68,42.56)	0.000
Sex (ref. Male)						
Female	1.57	(1.11, 2.24)	0.012	1.62	(1.16, 2.27)	0.005
Marital Status (ref. Married)						
Separated/Divorced	2.89	(1.68, 4.98)	0.000	2.22	(1.37, 3.62)	0.002
Single	0.32	(0.16, 0.63)	0.001	0.61	(0.29, 1.28)	0.191
Widowed/Widower	2.19	(1.13, 4.22)	0.020	1.01	(0.52, 1.98)	0.978
Education (ref. primary or less)						
Secondary	0.39	(0.23, 0.67)	0.001	0.58	(0.35, 0.96)	0.035
College/University	0.40	(0.20, 0.81)	0.011	0.75	(0.36, 1.58)	0.451
Income last month in KSh. (ref. <1000)						
>=1,000 to <10,000	0.70	(0.40, 1.24)	0.215	1.08	(0.61, 1.94)	0.782
>=10,000 to <20,000	0.59	(0.31, 1.12)	0.105	1.16	(0.60, 2.24)	0.658
>20,000	0.57	(0.27, 1.19)	0.131	1.18	(0.52, 2.71)	0.689

*ref =reference group

The prevalence of multimorbidity was higher among individuals who reported current alcohol use (aPR, 1.68 [95% CI, 1.07–2.64]; P=0.024) and ever alcohol use (aPR, 1.65 [95%CI, 1.04-2.62]; P=0.034). Although there were no clear associations between ever polydrug and multimorbidity, we observed an increased risk among participants who reported ever used 2 substances (aPR, 1.86 [95% CI, 1.20-2.90]; P=0.006) and among those who reported 3 or more substances (aPR,

1.06 [95% CI, 0.48-2.32]; P=0.890), albeit with low statistical power (Table 5). Ever or currently smoking cigarettes/ tobacco was not significantly associated with multi-morbidity (Table 5).

Table 5: Association between substance use and multimorbidity among the study participants.

	Model 1: Univariable Analysis			Model 2: Multivariable Analysis		
	PR	95% CI	p-value	PR	95% CI	p-value
Currently on any substance (ref. No)						
Yes	0.90	(0.57, 1.42)	0.655	1.14	(0.71, 1.83)	0.591
Ever used any substance (ref. No)						
Yes	1.27	(0.84, 1.94)	0.255	1.58	(0.98, 2.55)	0.059
Ever used multiple substances (ref. No)						
Yes	0.78	(0.49, 1.24)	0.297	0.76	(0.36, 1.62)	0.477
Currently using multiple substances (ref. No)						
Yes	0.69	(0.30, 1.55)	0.360	1.09	(0.44, 2.66)	0.857
Ever used multiple substances (ref. <2)						
2	1.67	(1.03, 2.70)	0.038	1.86	(1.20, 2.90)	0.006
3 or more	0.96	(0.57, 1.60)	0.863	1.06	(0.48, 2.32)	0.890
Currently using multiple substances (ref. <2)						
2	1.07	(0.64, 1.76)	0.802	1.14	(0.67, 1.93)	0.631
3 or more	0.69	(0.31, 1.57)	0.376	1.14	(0.47, 2.78)	0.771
Currently smoke cigarettes (ref. No)						
Yes	0.80	(0.41, 1.54)	0.491	0.76	(0.36, 1.63)	0.481
Currently smoke Cigarettes or tobacco (ref. <2)						
2 or more	0.91	(0.59, 1.43)	0.690	0.81	(0.37, 1.77)	0.592
Ever taken alcohol (ref. No)						
Yes	1.37	(0.92, 2.04)	0.119	1.65	(1.04, 2.62)	0.034
Currently taking alcohol (ref. No)						
Yes	1.11	(0.68, 1.82)	0.672	1.68	(1.07, 2.64)	0.024

*ref= reference group

Model 1 presents results from the univariable modified Poisson regression model, and Model 2 presents results from a multivariable modified Poisson regression model. In the multivariable analyses, each substance use variable has been adjusted for age, sex, marital status, education, and income.

Discussion

We found a relatively low prevalence of multimorbidity (7%) in our study compared to a global pooled estimate of 37% reported in a recent meta-analysis. The meta-analysis reported a wide range in multimorbidity prevalence, from 4% to 98%, with the African region showing the lowest rates among all regions(39). This lower prevalence in Africa has been attributed to a younger population and potential underdiagnosis of chronic conditions. In our study, the lower prevalence may also be partly explained by the limited number of chronic diseases included in the analysis. Multimorbidity in our study was associated with age, sex, marital status, and level of education. Significant risk factors identified for multimorbidity were older age (46+), being female, having a marital status of separated or divorced, and having a current history of alcohol use. Having a secondary level of education was found to be protective.

The strong association seen between advancing age and multimorbidity aligns with findings from other studies on multimorbidity trends(40–42). Biological aging has been considered a major risk factor for most chronic diseases, as the accumulation of cellular damage over time leads to a progressive decline in physiological integrity and impaired functions(43). Key mechanisms driving this cellular damage include genomic instability(44,45), telomere shortening(46), and mitochondrial dysfunction(47). Gaining deeper insights into these processes is crucial for developing strategies and interventions to slow the aging process. Additionally, there is a pressing need to design targeted health care strategies specifically for older adults.

We observed a higher likelihood of multimorbidity among females, which may be partly attributed to hormonal differences and other reproductive health factors(48). For instance, menopause and reproductive duration influence endogenous estrogen exposure, which has been associated with an increased risk of cardiovascular disease(49), metabolic dysregulation(50,51), increased inflammation(52,53) and cognitive decline(54). Other studies suggest that the greater burden of comorbidity among women compared to men may stem from women's higher propensity to disclose their health conditions in self-reports and greater utilization of health care services(55,56). However, our study collected data from household settings rather than hospital populations, and chronic illnesses were mostly clinically assessed rather than self-reported. This

strengthens the argument that, beyond sociocultural influences, biological factors likely play a significant role in the observed gender disparity in multimorbidity.

Consistent with findings from other studies(57), we observed a heightened risk of multimorbidity among separated, divorced, and widowed individuals in the univariable analysis. Limited social networks have been identified as key risk factors for multimorbidity(58,59), with psychosocial stress, economic instability, or social isolation serving as potential mediators of this relationship. Relationship dissolution is widely regarded as one of the most significant interpersonal stressors, often leading to challenges such as financial strain, housing instability, shifts in social connections, and, in cases involving children, difficulties with co-parenting and care arrangements(60–62). Interestingly, our univariable analysis suggested a protective effect of being single; however, this effect was attenuated in the multivariable analysis, indicating the need for further exploration of this relationship.

In our study, having a secondary level education was associated with lower prevalence of multimorbidity (aPR = 0.58, p = 0.035), suggesting a potential protective effect. However, this protective effect was not sustained for post-secondary education in the multivariable analysis. Educational attainment likely influences health outcomes by promoting health literacy(63) which can improve health-seeking behaviors and access to preventive care. Education level may also serve as a proxy measure of socioeconomic status, which is often linked to better health resources and opportunities(64). Interestingly, higher socioeconomic status has also been associated with sedentary behavior, which could partially explain the diminished protective effect observed at higher education levels (64).

Our findings revealed significantly stronger associations for individuals who reported "ever taken alcohol" (aPR = 1.65, p = 0.034) and "currently taking alcohol" (aPR = 1.68, p = 0.024) with multimorbidity, compared to weaker associations observed for the use of other substances or multiple substances. This highlights the unique and substantial impact of alcohol consumption on chronic disease development. Alcohol use is a well-documented risk factor for numerous health conditions, including hypertension, liver disease, cardiovascular disorders, and depression (9,65,66). The chronic and cumulative effects of alcohol can lead to systemic damage, increasing

the likelihood of multimorbidity over time. Additionally, alcohol consumption is often associated with lifestyle behaviors such as poor dietary choices, reduced physical activity, and smoking, which may further exacerbate health risks (67,68). The observed association underscores the critical importance of prioritizing alcohol use interventions as part of broader public health strategies aimed at reducing multimorbidity. Interventions such as screening, brief interventions, and referrals for treatment are effective in low-resource settings (69,70).

In contrast to findings from other studies (71,72), our analysis did not reveal significant associations between income levels and multimorbidity in the multivariable model. This lack of association may stem from several potential factors. First, there could be measurement limitations in how income was categorized or reported, such as income brackets that do not capture nuances of socioeconomic disparities or the influence of non-monetary wealth. Income data collected retrospectively or self-reported may also be subject to recall bias or underreporting. Second, cultural or contextual factors unique to the study population, such as informal support systems, may dampen the expected effect of income on multimorbidity. These findings suggest the need for further research to explore the complex interactions between income level and multimorbidity.

Our study's strengths include the robust sample size, which encompassed a diverse population from both urban and rural settings. This household-level survey utilized standardized random sampling procedures, minimizing selection biases and enhancing the generalizability of our findings. However, this study is not without limitations. Substance use measures were self-reported, which may have led to underreporting due to social desirability bias. Additionally, the reliance on self-reported data introduces the potential for recall bias. Furthermore, the cross-sectional design limits our ability to infer causality, as we can only report associations between factors rather than establish temporal or causal relationships. Lastly, the relatively limited number of diseases included in our disease pool may have contributed to a lower observed prevalence of multimorbidity. However, a key strength of our study lies in the inclusion of mental health conditions and the examination of substance use as a contributing factor to multimorbidity—an area often overlooked in similar research. Future studies could build on this work by incorporating

a broader range of chronic conditions, using longitudinal designs, and employing objective measures of both substance use and health status to enhance validity and generalizability.

Conclusion

Although the prevalence of multimorbidity in our study was lower than in comparable studies, it was positively associated with several sociodemographic factors, including older age group, female gender, and divorced or separated marital status, as well as clinical factors such as current alcohol use. These findings highlight the importance of addressing multimorbidity through a multidimensional approach that considers age, gender, and lifestyle factors while avoiding fragmented models of care. Tailored health interventions are particularly needed for older adults and women. Community-level strategies that target alcohol use and provide mental health support for divorced or widowed individuals are also recommended. Notably, higher levels of education appeared to be protective, underscoring the potential of educational initiatives to enhance health literacy and promote early preventive care.

Acknowledgement

We gratefully acknowledge David Mathu and Rodgers Ochieng for their dedicated coordination of field activities. We also thank the study research assistants for their invaluable support, and the team from the Kenya National Bureau of Statistics for their contributions to sampling, household selection, and data weighting. Finally, we extend our sincere appreciation to all study participants for their time and willingness to contribute to this research.

References

1. Harrison C, Fortin M, van den Akker M, Mair F, Calderon-Larranaga A, Boland F, et al. Comorbidity versus multimorbidity: Why it matters. J Multimorb Comorbidity. 2021;11:2633556521993993.

2. Skou ST, Mair FS, Fortin M, Guthrie B, Nunes BP, Miranda JJ, et al. Multimorbidity. Nat Rev Dis Primer. 2022 Jul 14;8(1):48.

3. Combating-Noncommunicable-Diseases-in-Kenya-An-Investment-Case.pdf [Internet]. [cited 2024 Nov 26]. Available from: https://documents1.worldbank.org/curated/en/428881586197529642/pdf/Combating-Noncommunicable-Diseases-in-Kenya-An-Investment-Case.pdf

4. Ammoun R, Wami WM, Otieno P, Schultsz C, Kyobutungi C, Asiki G. Readiness of health facilities to deliver non-communicable diseases services in Kenya: a national cross-sectional survey. BMC Health Serv Res. 2022 Aug 2;22(1):985.

5. Ashigbie PG, Rockers PC, Laing RO, Cabral HJ, Onyango MA, Buleti JPL, et al. Availability and prices of medicines for non-communicable diseases at health facilities and retail drug outlets in Kenya: a cross-sectional survey in eight counties. BMJ Open. 2020 May 15;10(5):e035132.

6. Onyango MA, Vian T, Hirsch I, Salvi DD, Laing R, Rockers PC, et al. Perceptions of Kenyan adults on access to medicines for non-communicable diseases: A qualitative study. PloS One. 2018;13(8):e0201917.

7. Onyango EM, Onyango BM. The Rise of Noncommunicable Diseases in Kenya: An Examination of the Time Trends and Contribution of the Changes in Diet and Physical Inactivity. J Epidemiol Glob Health. 2018 Dec;8(1–2):1–7.

8. Odeny L, Gathecha G, Mwenda V, Kendagor A, Cheburet S, Mugi B, et al. Tobacco smoking-attributable mortality in Kenya, 2012–2021. Tob Induc Dis. 2024 Jul 24;22(July):1–11.

9. GBD 2016 Alcohol and Drug Use Collaborators. The global burden of disease attributable to alcohol and drug use in 195 countries and territories, 1990-2016: a systematic analysis for the Global Burden of Disease Study 2016. Lancet Psychiatry. 2018 Dec;5(12):987–1012.

10. Mkuu RS, Gilreath TD, Barry AE, Nafukho FM, Rahman J, Chowdhury MAB, et al. Identifying individuals with multiple non-communicable disease risk factors in Kenya: a latent class analysis. Public Health. 2021 Sep 1;198:180–6.

11. Imtiaz S, Shield KD, Roerecke M, Samokhvalov AV, Lönnroth K, Rehm J. Alcohol consumption as a risk factor for tuberculosis: meta-analyses and burden of disease. Eur Respir J. 2017 Jul 13;50(1):1700216.

12. Alcoholism Fueling Spread of Tuberculosis In The Country | NACADA -National Authority for the Campaign Against Alcohol and Drug Abuse [Internet]. [cited 2024 Nov 28]. Available from: https://nacada.go.ke/alcoholism-fueling-spread-tuberculosis-country

13. Arenas DJ, Beltran S, Zhou S, Goldberg LR. Cocaine, cardiomyopathy, and heart failure: a systematic review and meta-analysis. Sci Rep. 2020 Nov 13;10(1):19795.

14. Kuussaari K, Karjalainen K, Niemelä S. Mental health problems among clients with substance use problems: a nationwide time-trend study. Soc Psychiatry Psychiatr Epidemiol. 2020 Apr 1;55(4):507–16.

15. Tolley A, Grewal K, Weiler A, Papameletiou AM, Hassan R, Basu S. Factors influencing adherence to non-communicable disease medication in India: secondary analysis of cross-sectional data from WHO - SAGE2. Front Pharmacol. 2023 Oct 13;14:1183818.

16. David RE, Ferrara KG, Schrecker J, Paculdo D, Johnson S, Bentley-Lewis R, et al. Impact of medication nonadherence and drug-drug interaction testing on the management of primary care patients with polypharmacy: a randomized controlled trial. BMC Med. 2024 Nov 18;22:540.

17. Cadri A, Nagumsi BAA, Twi-Yeboah A, Yeboah LD, Adomah-Afari A, Ane-Loglo MG, et al. Facilitators and Barriers to Health Seeking among People Who Use Drugs in the Sunyani Municipality of Ghana: An Exploratory Study. BioMed Res Int. 2021;2021(1):2868953.

18. Schuckit MA. Comorbidity between substance use disorders and psychiatric conditions. [cited 2024 Dec 13]; Available from: https://onlinelibrary.wiley.com/doi/10.1111/j.1360-0443.2006.01592.x

19. Stein DJ, Benjet C, Gureje O, Lund C, Scott KM, Poznyak V, et al. Integrating mental health with other non-communicable diseases. BMJ. 2019 Jan 28;364:l295.

20. Mohamed SF, Mwangi M, Mutua MK, Kibachio J, Hussein A, Ndegwa Z, et al. Prevalence and factors associated with pre-diabetes and diabetes mellitus in Kenya: results from a national survey. BMC Public Health. 2018 Nov 7;18(Suppl 3):1215.

21. Mohamed SF, Mutua MK, Wamai R, Wekesah F, Haregu T, Juma P, et al. Prevalence, awareness, treatment and control of hypertension and their determinants: results from a national survey in Kenya. BMC Public Health. 2018 Nov 7;18(Suppl 3):1219.

22. WHO-MSD-MER-2017.2-eng.pdf [Internet]. [cited 2024 Dec 8]. Available from: https://iris.who.int/bitstream/handle/10665/254610/WHO-MSD-MER-2017.2-eng.pdf?sequence=1

23. Nyongesa MK, Mwangi P, Wanjala SW, Mutua AM, Newton CRJC, Abubakar A. Prevalence and correlates of depressive symptoms among adults living with HIV in rural Kilifi, Kenya. BMC Psychiatry. 2019 Nov 1;19(1):333.

24. Ongeri L, McCulloch CE, Neylan TC, Bukusi E, Macfarlane SB, Othieno C, et al. Suicidality and associated risk factors in outpatients attending a general medical facility in rural Kenya. J Affect Disord. 2018 Jan 1;225:413–21.

25. Kwobah E, Epstein S, Mwangi A, Litzelman D, Atwoli L. PREVALENCE of psychiatric morbidity in a community sample in Western Kenya. BMC Psychiatry. 2017 Jan 18;17(1):30.

26. Degenhardt L, Charlson F, Ferrari A, Santomauro D, Erskine H, Mantilla-Herrara A, et al. The global burden of disease attributable to alcohol and drug use in 195 countries and territories, 1990–2016: a

systematic analysis for the Global Burden of Disease Study 2016. Lancet Psychiatry. 2018 Dec 1;5(12):987–1012.

27. National Survey on the Status of Drugs and Substance Use in Kenya 2022.pdf [Internet]. [cited 2024 Dec 8]. Available from: https://nacada.go.ke/sites/default/files/2023-05/National%20Survey%20on%20the%20Status%20of%20Drugs%20and%20Substance%20Use%20in%20Kenya%202022.pdf

28. McHugh RK, Votaw VR, Sugarman DE, Greenfield SF. Sex and Gender Differences in Substance Use Disorders. Clin Psychol Rev. 2018 Dec;66:12–23.

29. Vasilenko SA, Evans-Polce RJ, Lanza ST. Age trends in rates of substance use disorders across ages 18–90: Differences by gender and race/ethnicity. Drug Alcohol Depend. 2017 Nov 1;180:260–4.

30. Ford JA, Pomykacz CR, Ortiz K, McCabe SE, Schepis TS. Educational attainment and prescription drug misuse: The importance of push and pull factors for dropping out. J Crim Justice. 2020 Jan 1;66:101636.

31. Fleming CB, White HR, Catalano RF. Romantic Relationships and Substance Use in Early Adulthood: An Examination of the Influences of Relationship Type, Partner Substance Use, and Relationship Quality. J Health Soc Behav. 2010 Jun;51(2):153–67.

32. Baptiste-Roberts K, Hossain M. Socioeconomic Disparities and Self-reported Substance Abuse-related Problems. Addict Health. 2018 Apr;10(2):112–22.

33. 2019 Kenya Population and Housing Census Results - Kenya National Bureau of Statistics [Internet]. 2019 [cited 2024 Dec 30]. Available from: https://www.knbs.or.ke/2019-kenya-population-and-housing-census-results/

34. Jung SH. Stratified Fisher's Exact Test and its Sample Size Calculation. Biom J Biom Z. 2014 Jan;56(1):10.1002/bimj.201300048.

35. Stephanie. Statistics How To. 2017 [cited 2023 Jun 28]. Kish Grid: What It Is and How to Use It. Available from: https://www.statisticshowto.com/kish-grid/

36. Kroenke K, Spitzer RL, Williams JB. The PHQ-9: validity of a brief depression severity measure. J Gen Intern Med. 2001 Sep;16(9):606–13.

37. Dept WHOMH and SD, WHO Initial Consultation Group Meeting on Preparation of a Basic Manual on Substance Abuse Epidemiology (1992 : Geneva S, Meeting on the Consolidated Epidemiological Manual on Substance Abuse (1994 : Athens G. Guide to drug abuse : epidemiology. 2000 [cited 2025 Feb 19]; Available from: https://iris.who.int/handle/10665/63850

38. StataCorp. Stata Statistical Software. College Station, TX: StataCorp LLC; 2023.

39. Chowdhury SR, Das DC, Sunna TC, Beyene J, Hossain A. Global and regional prevalence of multimorbidity in the adult population in community settings: a systematic review and meta-analysis. eClinicalMedicine [Internet]. 2023 Mar 1 [cited 2025 Apr 8];57. Available from: https://www.thelancet.com/journals/eclinm/article/PIIS2589-5370(23)00037-8/fulltext

40. van den Akker M, Buntinx F, Metsemakers JF, Roos S, Knottnerus JA. Multimorbidity in general practice: prevalence, incidence, and determinants of co-occurring chronic and recurrent diseases. J Clin Epidemiol. 1998 May;51(5):367–75.

41. Marengoni A, Angleman S, Melis R, Mangialasche F, Karp A, Garmen A, et al. Aging with multimorbidity: a systematic review of the literature. Ageing Res Rev. 2011 Sep;10(4):430–9.

42. Salisbury C, Johnson L, Purdy S, Valderas JM, Montgomery AA. Epidemiology and impact of multimorbidity in primary care: a retrospective cohort study. Br J Gen Pract J R Coll Gen Pract. 2011 Jan;61(582):e12-21.

43. Fabbri E, Zoli M, Gonzalez-Freire M, Salive ME, Studenski SA, Ferrucci L. Aging and Multimorbidity: New Tasks, Priorities, and Frontiers for Integrated Gerontological and Clinical Research. J Am Med Dir Assoc. 2015 Aug 1;16(8):640–7.

44. Laffon B, Bonassi S, Costa S, Valdiglesias V. Genomic instability as a main driving factor of unsuccessful ageing: Potential for translating the use of micronuclei into clinical practice. Mutat Res Mutat Res. 2021 Jan 1;787:108359.

45. Vijg J, Suh Y. Genome instability and aging. Annu Rev Physiol. 2013;75:645–68.

46. Rossiello F, Jurk D, Passos JF, d'Adda di Fagagna F. Telomere dysfunction in ageing and age-related diseases. Nat Cell Biol. 2022 Feb;24(2):135–47.

47. Guo Y, Guan T, Shafiq K, Yu Q, Jiao X, Na D, et al. Mitochondrial dysfunction in aging. Ageing Res Rev. 2023 Jul 1;88:101955.

48. Burden and determinants of multimorbidity ... | Wellcome Open Research [Internet]. [cited 2025 Jan 3]. Available from: https://wellcomeopenresearch.org/articles/5-275

49. Zhu D, Chung HF, Dobson AJ, Pandeya N, Giles GG, Bruinsma F, et al. Age at natural menopause and risk of incident cardiovascular disease: a pooled analysis of individual patient data. Lancet Public Health. 2019 Nov;4(11):e553–64.

50. Jeong HG, Park H. Metabolic Disorders in Menopause. Metabolites. 2022 Oct 8;12(10):954.

51. Mauvais-Jarvis F, Clegg DJ, Hevener AL. The role of estrogens in control of energy balance and glucose homeostasis. Endocr Rev. 2013 Jun;34(3):309–38.

52. Khalafi M, Malandish A, Rosenkranz SK. The impact of exercise training on inflammatory markers in postmenopausal women: A systemic review and meta-analysis. Exp Gerontol. 2021 Jul 15;150:111398.

53. Abildgaard J, Tingstedt J, Zhao Y, Hartling HJ, Pedersen AT, Lindegaard B, et al. Increased systemic inflammation and altered distribution of T-cell subsets in postmenopausal women. PloS One. 2020;15(6):e0235174.

54. Hao W, Fu C, Dong C, Zhou C, Sun H, Xie Z, et al. Age at menopause and all-cause and cause-specific dementia: a prospective analysis of the UK Biobank cohort. Hum Reprod Oxf Engl. 2023 Sep 5;38(9):1746–54.

55. Alimohammadian M, Majidi A, Yaseri M, Ahmadi B, Islami F, Derakhshan M, et al. Multimorbidity as an important issue among women: results of a gender difference investigation in a large population-based cross-sectional study in West Asia. BMJ Open. 2017 May 1;7(5):e013548.

56. Sembiah S, Dasgupta A, Taklikar CS, Paul B, Bandyopadhyay L, Burman J, et al. Gender inequalities in prevalence, pattern and predictors of multimorbidity among geriatric population in rural West Bengal. J Fam Med Prim Care. 2022 Aug 30;11(8):4555.

57. Pellón-Elexpuru I, Van Dijk R, Van der Valk I, Martínez-Pampliega A, Molleda A, Cormenzana S. Divorce and physical health: A three-level meta-analysis. Soc Sci Med. 2024 Jul 1;352:117005.

58. Wang D, Li D, Mishra SR, Lim C, Dai X, Chen S, et al. Association between marital relationship and multimorbidity in middle-aged adults: a longitudinal study across the US, UK, Europe, and China. Maturitas. 2022 Jan 1;155:32–9.

59. Xu X, Mishra GD, Jones M. Evidence on multimorbidity from definition to intervention: An overview of systematic reviews. Ageing Res Rev. 2017 Aug 1;37:53–68.

60. Leopold T. Gender Differences in the Consequences of Divorce: A Study of Multiple Outcomes. Demography. 2018 Apr 13;55(3):769–97.

61. Zineldin M. TCS is to Blame: The Impact of Divorce on Physical and Mental Health. Int J Prev Med. 2019;10(1):141.

62. van Tilburg TG, Aartsen MJ, van der Pas S. Loneliness after Divorce: A Cohort Comparison among Dutch Young-Old Adults. Eur Sociol Rev. 2015 Jun 1;31(3):243–52.

63. Liu L, Qian X, Chen Z, He T. Health literacy and its effect on chronic disease prevention: evidence from China's data. BMC Public Health. 2020 May 14;20(1):690.

64. Rodriguez Roca B, Tully MA, Sansano-Nadal O, Caserotti P, Coll-Planas L, Roqué M, et al. Is education level, as a proxy for socio-economic position, related to device-measured and self-reported sedentary behavior in European older adults? A cross-sectional study from the SITLESS project. Front Public Health. 2023;11:1296821.

65. Parry C, Patra J, Rehm J. Alcohol consumption and non-communicable diseases: epidemiology and policy implications. Addict Abingdon Engl. 2011 Oct;106(10):1718–24.

66. Workplace NRC (US) and I of M (US) C on DU in the, Normand J, Lempert RO, O'Brien CP. Impact of Alcohol and Other Drug Use: Observational/Field Studies. In: Under the Influence? Drugs and the American Work Force [Internet]. National Academies Press (US); 1994 [cited 2023 Jun 28]. Available from: https://www.ncbi.nlm.nih.gov/books/NBK236258/

67. Satre DD, Gordon NP, Weisner C. Alcohol Consumption, Medical Conditions, and Health Behavior in Older Adults. Am J Health Behav. 2007;31(3):238–48.

68. Ma J, Betts NM, Hampl JS. Clustering of lifestyle behaviors: the relationship between cigarette smoking, alcohol consumption, and dietary intake. Am J Health Promot AJHP. 2000;15(2):107–17.

69. van der Westhuizen C, Malan M, Naledi T, Roelofse M, Myers B, Stein DJ, et al. Patient outcomes and experience of a task-shared screening and brief intervention service for problem substance use in South African emergency centres: a mixed methods study. Addict Sci Clin Pract. 2021 May 12;16(1):31.

70. Jaguga F, Ott MA, Kwobah EK, Apondi E, Giusto A, Barasa J, et al. Adapting a substance use screening and brief intervention for peer-delivery and for youth in Kenya. SSM - Ment Health. 2023 Dec 15;4:100254.

71. Pathirana TI, Jackson CA. Socioeconomic status and multimorbidity: a systematic review and meta-analysis. Aust N Z J Public Health. 2018 Apr 1;42(2):186–94.

72. Knies G, Kumari M. Multimorbidity is associated with the income, education, employment and health domains of area-level deprivation in adult residents in the UK. Sci Rep. 2022 May 4;12(1):7280.

Figure 1-Map of Kenya showing the various county boundaries and the study areas

Click here to access/download;Figure;Figure 1.tif

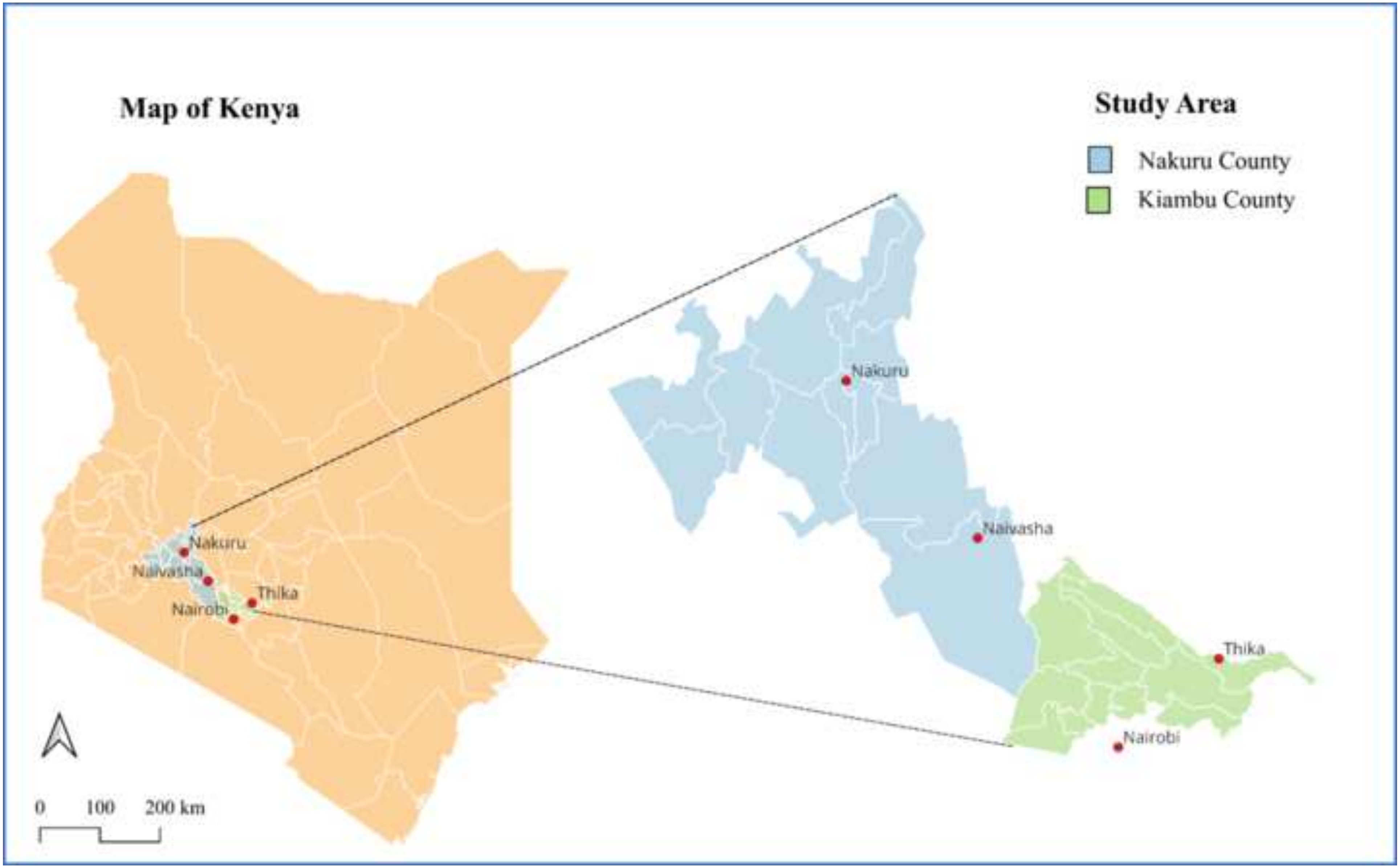

Figure 2-Frequency of unique combinations of chronic disease comorbidity

Click here to access/download;Figure;Figure 2.tif

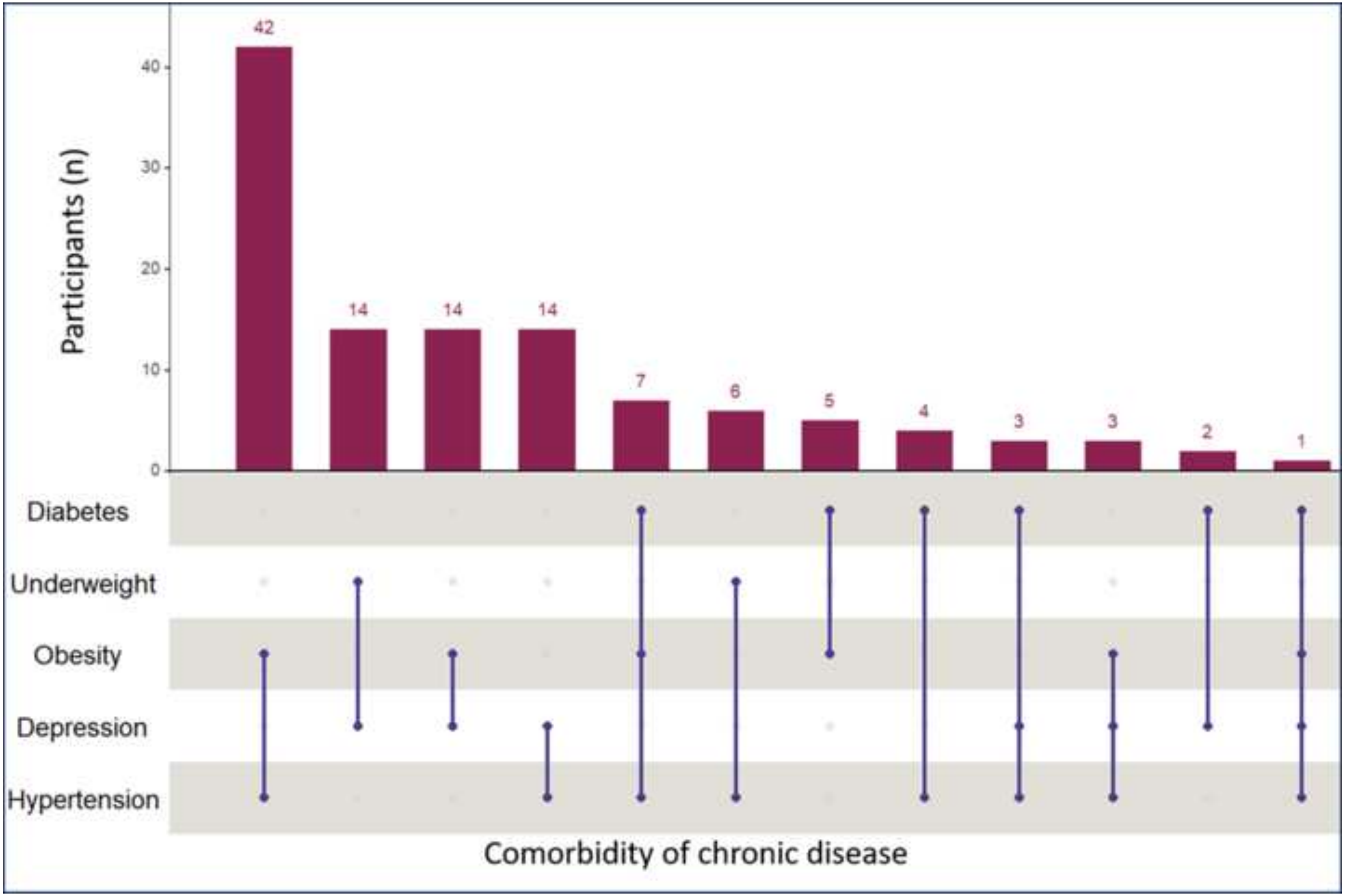

Figure 3-Prevalence of multimorbidity and chronic conditions by age

Click here to access/download;Figure;Figure 3.tif

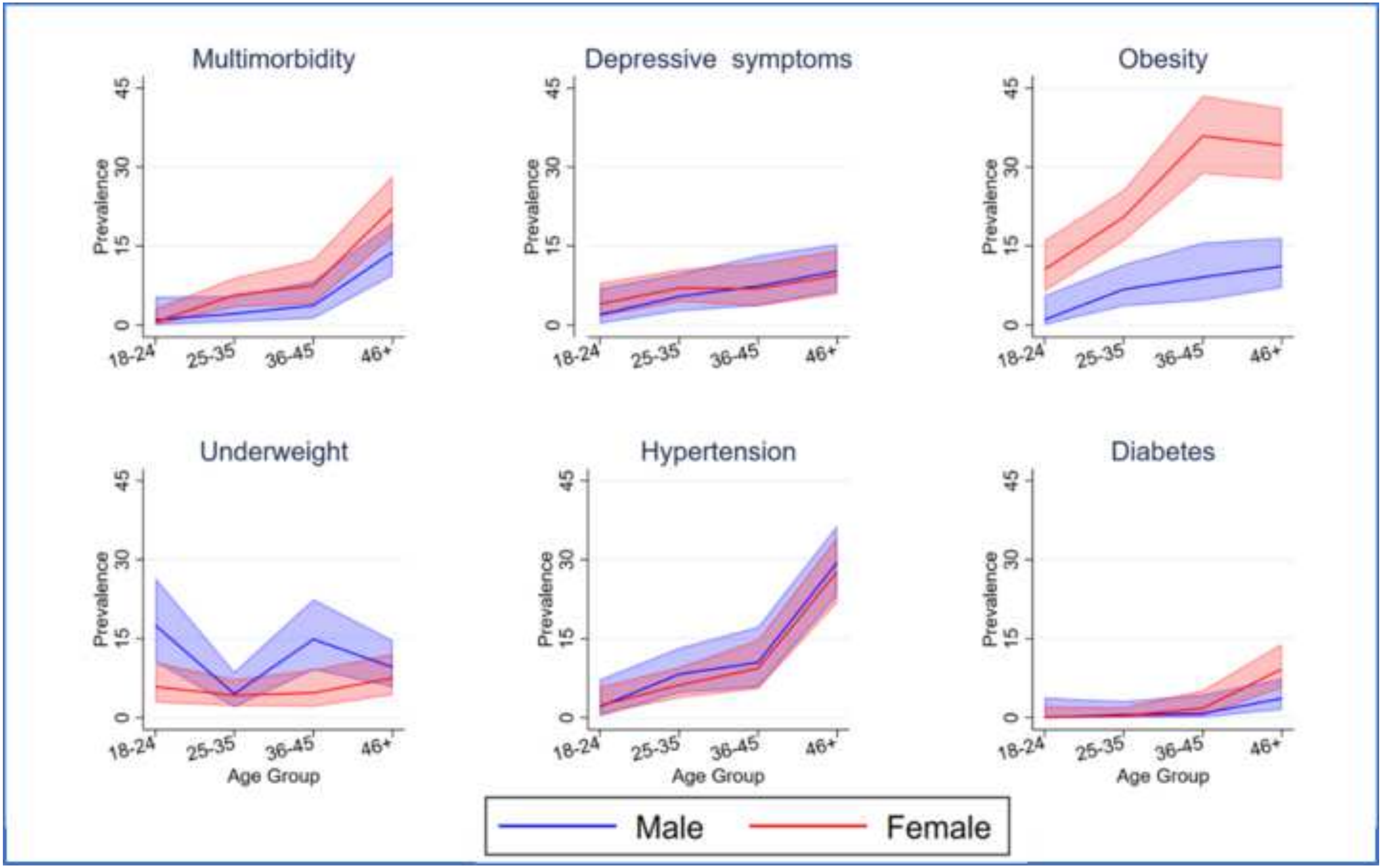

Supplement: Attachment — Submitted filename: PGPH-D-25-01204R1.pdf [file pgph.0005544.s002.pdf]
