# Supplementary material for: Socioeconomic inequalities, substance use, and chronic multimorbidity in Kiambu and Nakuru counties in Kenya
Source: PLOS Glob Public Health. 2025 Dec 8;5(12):e0005544. doi: 10.1371/journal.pgph.0005544 (PMC12685187; doi:10.1371/journal.pgph.0005544)
Supplement: Attachment — Submitted filename: Rebuttal Letter_14-10-25.docx [file pgph.0005544.s004.docx]

Editors’ requirement

1. We ask that a manuscript source file be provided at Revision. Please upload your manuscript file as a .doc, .docx, .rtf or .tex.

**Response: This has been done**.

2. Competing interest statement.

**Response: We have included a competing interest statement as follows: “The authors have declared that no competing interests exists.”**

3. In the online submission form, you indicated that “Data used in this analysis will be published online once all protocol objectives are completed. Reasonable requests for data can be directed to the corresponding author.” All PLOS journals now require all data underlying the findings described in their manuscript to be freely available to other researchers, either a. In a public repository, b. Within the manuscript itself, or c. Uploaded as supplementary information.

**Response: “Source data are provided in the supplementary materials (Multimorbidity.xlsx file). Source data are provided with this paper”**

4. Potential Copyright Issues: Figure 1: please (a) provide a direct link to the base layer of the map (i.e., the country or region border shape) and ensure this is also included in the figure legend; and (b) provide a link to the terms of use / license information for the base layer image or shapefile. We cannot publish proprietary or copyrighted maps (e.g. Google Maps, Mapquest) and the terms of use for your map base layer must be compatible with our CC-BY 4.0 license. Note: if you created the map in a software program like R or ArcGIS, please locate and indicate the source of the basemap shapefile onto which data has been plotted. If your map was obtained from a copyrighted source please amend the figure so that the base map used is from an openly available source. Alternatively, please provide explicit written permission from the copyright holder granting you the right to publish the material under our CC-BY 4.0 license. Please note that the following CC BY licenses are compatible with PLOS license: CC BY 4.0, CC BY 2.0 and CC BY 3.0, meanwhile such licenses as CC BY-ND 3.0 and others are not compatible due to additional restrictions. If you are unsure whether you can use a map or not, please do reach out and we will be able to help you. The following websites are good examples of where you can source open access or public domain maps: * U.S. Geological Survey (USGS) - All maps are in the public domain. (<http://www.usgs.gov>) * PlaniGlobe - All maps are published under a Creative Commons license so please cite “PlaniGlobe, ttp://www.planiglobe.com, CC BY 2.0” in the image credit after the caption. (<http://www.planiglobe.com/?lang=enl>) * Natural Earth - All maps are public domain. (http://www.naturalearthdata.com/about/terms-of-use/)

**Response**: Thank you for providing the details. In the methods section line 108-112, we have included the details in the graph legend as follows:

“Figure 1: Map of Kenya showing the various county boundaries and the study areas. Base layer sources: The Kenya administrative boundary Shape files were retrieved from the Humanitarian Data Exchange website (<https://data.humdata.org/dataset/cod-ab-ken>) under an open license (<https://www.google.com/search?q=https://data.humdata.org/about/license/>). Maps were generated using open-source QGIS version 3.34.2 (<https://qgis.org/>). ”

Response to Reviewer #1: R1: We thank the reviewer for their thoughtful and helpful comments.

1. Fine and clear topic, but needs great effort to be obvious

**Response: We thank the reviewer for raising this, and we have reworded the title to improve clarity. The title now reads “Socioeconomic inequalities, substance use, and chronic multimorbidity in Kiambu and Nakuru counties in Kenya.”**

2. Do not use pronouns in research language.

**Response: We respectfully acknowledge the reviewer’s comment regarding the use of pronouns such as “*we”*. However, we believe that the use of first-person pronouns in scientific writing is an accepted stylistic choice and is consistent with the conventions of many high-impact journals such as the PLOS series and others. These journals often use “*we”* to clearly indicate the authors' role in conducting the study, interpreting the findings, and drawing conclusions, thereby enhancing clarity and readability. Nevertheless, we are happy to modify instances where the use of “*we”* may appear unnecessary or informal, while retaining it in contexts where it improves precision and readability. For example paper “Dixon J, Dhodho E, Mundoga F, Webb K, Chimberengwa P, Mhlanga T, et al. Multimorbidity and health system priorities in Zimbabwe: A participatory ethnographic study. PLOS Glob Public Health. 2025 Apr 28;5(4):e0003643.”**

3. Use mean full and brief key words 3-5 words as maximum

**Response: We thank the reviewer and have added keywords as follows: “Keywords: Multimorbidity, Substance use, Alcohol, Smoking”**

4. The abstract is presented in a clear way, but some information was missed. Please give a short background in the abstract.

**Response: We have added a definition of multimorbidity in the abstract in addition to the already provided information on the burden and impact of multimorbidity.**

**Introduction**

5. Author can numerate the multi-morbidity disease in Kenya. Focus on multimorbidity of the current study.

**Response: Thank you for this helpful suggestion. We have revised the introduction section for better flow and to ensure the focus remains on multimorbidity of the current study. For example, line 90-93 reads “This population-based cross-sectional study conducted in two populous Kenyan counties investigates the relationship between multimorbidity—the co-occurrence of depression, hypertension, diabetes, obesity, or underweight—and its association with substance use and socioeconomic status.” Further, we have deleted information in the introduction about HIV as this measure was not included in our analysis**.

6. The end of the introduction should contain complete idea of study problem. The study problem should be similar to that of aim of the study in the abstract.

**Response: We thank the reviewer for this helpful comment. We have highlighted the study problem at the end of the introduction, line 87-89 now reads “Yet, despite these consistent findings from high- and middle-income countries, data from low-income settings in SSA remain scarce, leaving major gaps in understanding the association of substance use and social disadvantage with multimorbidity.”**

5. Avoid using pronouns like we

**Response: We respectfully acknowledge the reviewer’s comment regarding the use of pronouns such as “*we”*. However, we believe that the use of first-person pronouns in scientific writing is an accepted stylistic choice and is consistent with the conventions of many high-impact journals such as the PLOS series and others. These journals often use “*we”* to clearly indicate the authors' role in conducting the study, interpreting the findings, and drawing conclusions, thereby enhancing clarity and readability. Nevertheless, we are happy to modify instances where the use of “*we”* may appear unnecessary or informal, while retaining it in contexts where it improves precision and readability. For example paper “Dixon J, Dhodho E, Mundoga F, Webb K, Chimberengwa P, Mhlanga T, et al. Multimorbidity and health system priorities in Zimbabwe: A participatory ethnographic study. PLOS Glob Public Health. 2025 Apr 28;5(4):e0003643.”**

6. Methodology needs great effort to be acceptable. It contains some literature that contradicts the content of the methods. Rewrite with the specific titles, type of study, description of the participants, methods of collecting data, method of sample collection, inclusion and exclusion criteria, and dividing participants into groups according to study objects. Data analysis. Study outcomes are related to the expected results.

**Response: Thank you for this observation. We structured the Methods section in accordance with the journal’s template to ensure alignment. We confirm that all the information requested has been included within the Methods section, though presented as per the required format.**

7. Results: prefer to use a table beside text. Put every table next to its text.

**Response: Similarly, here we were following the journal’s guidance on presenting results.**

8. Discussion is fine but long, focus on main and significant results, divide the results from limitation, recommendations and conclusion. Conclusion is restricted towards the study object.

**Response: We have done some edits in the discussion, adding on reviewer 2 recommendations, which have greatly improved the clarity of the discussion. The formatting for the discussion and conclusion section was again based on the journal’s guidelines and template. The reviewer’s comments have been factored in in the current version.**

**Reviewer #2**

The authors have completed a population-based survey in two counties in Kenya and used the resulting data to explore the cross-sectional associations alcohol and other substances of abuse and multimorbidity. I congratulate the authors on the completion of the survey, a rare piece of important data from Kenya and across the region. The current analysis has a number of substantial limitations and interpretations require some rethinking.

**Response: We sincerely thank you for your thorough and comprehensive review, which has greatly contributed to improving the overall quality of the manuscript.**

1. The abstract lacks a definition for “substance use” and “multimorbidity” given that the precise definition is critical to understand the results, these need to be clearly defined here.

**Response: Thank you for this suggestion. We have defined both substance use and multimorbidity within the abstract. Line 23-27 now reads “**Multimorbidity, defined as the coexistence of two or more chronic conditions in an individual, is a growing public health concern associated with increased healthcare costs, poorer quality of life, and increased mortality. Substance use, defined as the consumption of alcohol, tobacco, or other psychoactive substances, may influence the development of multimorbidity, but evidence from sub-Saharan Africa remains limited.**”**

2. The abstract does not specify whether the estimates shared come from univariate or multivariate models

**Response: We have now specified that the findings are from the multivariate models. Line 30 to 33 now reads “To account for the complex survey design, we employed sample-weighted multivariable analysis using a modified Poisson regression model to estimate adjusted prevalence ratios (aPR) and 95% confidence intervals (CIs) for the associations.”**

3. The abstract uses the term “female gender” but gender was not measured by the authors. The term is female.

**Response: We agree and have revised sections that read female gender to read female sex.**

4. The proportion of married individuals was misstated as 60% when in the text it was reported to be 61%

**Response: We have now revised line 33 of the abstract to read 61%.**

5. On line 52 the authors write “Multimorbid conditions focus on a holistic…” Conditions don’t “focus” rather the users of the multimorbidity concept may “focus.”

**Response: Indeed, we agree with the reviewer. For clarity we have revised the section in the introduction, line 56-59 now reads “**Whilst single conditions can be managed with standardized, vertical programs, multimorbidity demands integrated, patient-centered approaches that account for interactions among multiple conditions, social determinants of health, and behavioral risk factors (1).”

6. The authors claim based on reference 2 that multimorbidity is growing, which presumably meets the rates are increasing. Reference 2 does not make that claim.

**Response: We have corrected this and cited the correct reference** “Chowdhury SR, Chandra Das D, Sunna TC, Beyene J, Hossain A. Global and regional prevalence of multimorbidity in the adult population in community settings: a systematic review and meta-analysis. eClinicalMedicine. 2023 Feb 16;57:101860”

7. The use of the word “carry” on liner 57 is incorrect.

**Response: We have replaced carry with the word account for and restructured the sentence for more clarity, line 74 of the introduction now reads. “NCDs account for over 30% of deaths nationally and contribute significantly to rising DALYs.**

8. There are 2 full pages of introduction and much of the material is not directed at the subject at hand. The background should be shortened and focus only on the prevalence and predictors of the examined multimorbid conditions, and it should be restricted mainly to Kenya. For example, there is no need to give special consideration to people living with HIV as that is not what this paper aims to explore.

**Response: We have removed text on HIV and patient population as we did not specifically examine this in our study. We have also cut down the introduction to reflect Multimorbidity as our key outcome measure.**

9. Line 85 refers to the prevalence of depression in patient populations, but it isn’t clear what kind of patients they are discussing.

**Response: We have edited out this section for relevance as guided by the earlier comment.**

10. On line 135 they say that the survey was limited to subjects that spoke English or Kiswahili. They should share the fraction of subjects that were excluded on this basis.

**Response: We thank the reviewer for this observation. While we did not collect precise data on the number of individuals excluded due to language, this was a very small fraction. The study counties border Nairobi and are highly urbanized, with the majority of residents conversant in either English or Kiswahili. We have clarified this point in the methods section, line 118-120 now reads.** **“Only a very small number of potential participants were excluded due to language barriers, as the study counties border Nairobi and the vast majority of residents are conversant in either English or Kiswahili.”**

11. On line 157 they say that they took actions to minimize non-response bias, but they don’t report the response rate. It should be here.

**Response: We have now included the response rate in our results. Line 224-225 reads “A total of 1,484 study participants were enrolled, with response rates of 72.9% in Kiambu and 76.9% in Nakuru.”**

12. On lines 172-177 they describe data reclassification, but they have yet to explain where that data would come from, so the methods are mis-ordered. Furthermore, they describe classifying fasting blood sugars, but I don’t know where that data would come from as on line 182 they say they assesses “Random Blood Sugar” levels but there is no indication how that testing was completed.

**Response. We have included further detail as follows from line 153-162: “**Weight was measured to the nearest 0.1 kg using a calibrated digital weighing scale (SECA® 803), and height was measured to the nearest 0.1 cm using a portable stadiometer (SECA® 213), following standardized procedures. Body mass index (BMI) was calculated as weight in kilograms divided by the square of height in meters. Nutritional status was classified as obese (BMI ≥30) or underweight (BMI <18.5). Blood pressure was measured on the right arm using a validated Omron M6 automated device after participants had rested for at least 5 minutes and abstained from smoking, caffeine, or other stimulants. Three measurements were taken at 5-minute intervals, and the mean value was used for analysis. Random blood glucose was measured from a finger-prick blood sample using a portable point-of-care glucometer (Accu-Chek® Active, Roche Diagnostics), following standardized procedures.

13. The definition of multimorbidity given on lines 198-199 needs to be in the abstract

**Response: This has now been included. “**Multimorbidity, defined as the coexistence of two or more chronic conditions in an individual, … **”**

14. On line 220 in the methods, the authors claim they use a backwards stepwise model, but I see no evidence of this in the results (e.g. there is nothing about that in Table 4).

**Response; We thank the reviewer for noting this oversight. The table based on this methodology was removed during revisions, but the corresponding statement in the statistical analysis section was inadvertently left in place.**

15. On lines 242-244 they detail the prevalence of the underlying conditions that are summed to make multimorbidity only after they have reported the summed variable prevalence. That is out of order.

**Response: We thank the reviewer and reorganized the sentences for clarity. Line 232-235 now reads “**Among the five chronic conditions assessed, 17.7% (n = 250) of participants were obese, 12.6% (n = 183) had hypertension, 7.6% (n = 108) were underweight, 7.0% (n = 104) reported depressive symptoms, and 2.2% (n = 31) had diabetes. Overall, 7.7% (n = 115) met criteria for multimorbidity (≥2 conditions), and only one participant had four conditions.**”**

16. In Tabel 1, the top age range is described as 46+, that is not enough information. The actual range and the mean age in that range need to be shared somewhere in the paper.

**Response: We have added more detail in the table legend as follows “Among participants aged 46 years and older, the age ranged from 46 to 99 years, with a median (IQR) of 58 (51–69) years.”**

17. Table 2 uses many different ways of saying “using” a substance. All of these should be converted to use, not “on” or “taking”.

**Response: We thank the reviewer for the attention to detail. We have changed that to “History of substance use and current substance use.”**

18. The authors don’t explain what forms of tobacco are used other than cigarettes, and the phrase “cigarettes or tobacco” is confusing since cigarettes are tobacco products.

**Response: We thank the reviewer and have added text to differentiate between smoking and other uses of tobacco, that is, smoking cigarettes, chewing/sniffing tobacco. We have specified that in the Statistical analysis section, line 215-2158 reads** “We conducted sensitivity analyses to examine the association between multimorbidity and a history of substance use, defined as either current use or previous use among individuals who had stopped substance use, potentially for reasons such as medical advice, personal choice, or health concerns.” **and tables as follows: “Current tobacco use (smoke cigarettes or chew/sniff tobacco)”**

19. Testing and presenting correlations between a summed variable (multimorbidity) and the underlying conditions does not make sense. They should describe the prevalence of each, but a correlation is not justified.

**Response: We thank the reviewer for the comments and have deleted the entire correlation table and the narrative within the results section.**

20. The statement on Line 277 makes no sense, by definition current users are ever users. I wonder what they were trying to convey and if they understand their data?

**Response: We appreciate the reviewer’s comment and agree that, by definition, *current users* are indeed a subset of *ever users*. We intended to clarify that the category *ever users* included both *current users* and *former users*—that is, individuals who previously used the substance but had since stopped, often for reasons such as medical advice, personal choice, or health concerns. We have revised the text to make this distinction explicit and avoid confusion. Please see line 215-218. Whilst the main objective was to assess current alcohol use, we included ever use as a sensitivity analysis. “**In the analysis of substance use, the likelihood of multimorbidity was significantly higher among individuals who reported current alcohol use (aPR 1.68; 95% CI, 1.07–2.64; P=0.024) and was consistent in a sensitivity analysis that examined a history of alcohol use (aPR 1.65; 95% CI, 1.04–2.62; P=0.034) regardless of current use.**”**

21. On line 278, they use the word interesting which is conversational, an editorial comment, and does not belong in the results section of the paper.

**Response: This has been deleted from the results section.**

22. Table 3 is in fact a figure.

**Response; This has been deleted from the analyses based on an earlier suggestion by another reviewer.**

23. The authors have not completed the proper test of association for education. To test for an education effect, they need to conduct a 2 df test on the association of the two categories with the outcome and only that can determine if there is an overall education effect.

**Response: We thank the reviewer for highlighting this point. We have now performed the appropriate 2 degrees of freedom Wald test to assess the overall association between education and multimorbidity in the multivariable model using survey weights. The global p-value for education was 0.1043, indicating no statistically significant overall association. We have updated the Statistical Methods and Results accordingly. Line 281-282 now reads “**Moreover, there was no evidence of an overall association between multimorbidity and either education level (Wald test, 2 df; P=0.1043) or income in the past month (Wald test, 3 df; P=0.9657) (Table 3).**”**

24. The authors make the claim on page 14/15 that there is an association among those using 3 or more substances, but the P is 0.89. That means there is nothing there.

**Response; This has been corrected**

25. In the second paragraph of the discussion, the authors make odd statements about what biological aging is. This has nothing to do with their work and does not belong in the paper. And there is no such thing as “slowing the aging process.”

**Response: This statement has been rewritten to refer to potential reasons why older groups may be more susceptible to multimorbidity compared to younger age groups. Line 312- 316 now reads “**The strong association between age and multimorbidity observed in our study is consistent with findings from diverse populations, where the prevalence of chronic multimorbidity is markedly higher in older age groups (5,23). This pattern may reflect cumulative exposure to risk factors across the life course, coupled with declining physiologic reserve and increased vulnerability to chronic diseases in later life (24). **”**

26. The authors again use the word gender on page 17 but haven’t measured gender.

**Response: This has been changed to sex. Line 319-320 reads “**We also observed sex differences consistent with prior studies, in which women bear a greater burden of multimorbidity than men. The higher prevalence of multimorbidity among women persisted even after adjusting for age in regression models, suggesting that factors beyond life expectancy.**”**

27. The authors make an incorrect claim about the effect of being widowed. There is no association with being widowed in the full model.

**Response: This has been corrected in line 326 -328 the statement now reads “**However, never being married was not associated with higher multimorbidity in our multivariable analyses, consistent with a recent nationally representative study in South Africa.**”**

28. The authors use reference 64 to make very speculative claims about the educational effects that are not warranted.

**Response: In line 329-339, we have edited this paragraph as follows “In contrast with studies in some settings(15,31), we found no consistent associations between multimorbidity and income or educational attainment. Several factors may explain this finding. First, the high unemployment rates in Kenya across all education levels may weaken the expected link between education, income, and health, as income in the past month may not fully capture long-term socioeconomic position or material deprivation (32). Second, widespread informal support systems may buffer the direct effects of low income on health outcomes, further attenuating observed associations (33). Third, studies spanning high-, middle-, and low-income countries have reported a weaker association between multimorbidity and socioeconomic indicators in low-income countries (34). This suggests that the strength of this relationship is highly context-specific, indicating that the strength and direction of these associations may be context-specific.”**

29. The authors claim that their data shows that alcohol has a “unique and substantial impact on chronic disease development” on page 17. That is not correct. Their data can be used to apportion cross-sectional associations in their population but not make grand claims about all the other substances of abuse.

**Response; Thank you for your comment. We have revised the text to clarify that our findings indicate an association between alcohol use and multimorbidity in this population. We have removed any language implying a unique or causal impact, as our cross-sectional data only support observed associations rather than definitive conclusions about alcohol relative to other substances. Line 344-347 now reads “The significant association between alcohol use and multimorbidity in this population underscores the potential value of integrating alcohol screening and interventions into primary care, particularly in low-resource settings where preventive services are limited.”**

30. The authors seem to misinterpret the role of multivariate models at the top of page 18 as they describe confounding effects of tobacco etc on alcohol consumers. The point of the models was to separate those effects, so why revert to describing it this way?

**Thank you for pointing this out. We have revised the text to accurately reflect the analyses that were conducted. Line 341-342 reads “**Our analysis of behavioral risk factors revealed an association between current alcohol use and multimorbidity, whereas tobacco and other substance use showed no significant associations.**”**

31. They go on to speculate about the need for “brief interventions” and treatment despite discussing ever use of alcohol! Ever users of alcohol do not need interventions or treatment. They haven’t assessed Alcohol Use Disorder, which might suggest what needs to be done to improve population health.

**Response: We have revised this section to clarify that both previous and current alcohol use are considered important risk factors for multimorbidity in the regression model. We have removed any implication that all individuals with prior alcohol use require interventions or treatment, as this was not assessed in our study. Line 344-347 reads; “The significant association between alcohol use and multimorbidity in this population highlights the potential value of integrating alcohol screening and interventions into primary care, particularly in low-resource settings where preventive services are limited.”**

32. The conclusion makes a claim about “comparable studies,” but I don’t know what that is referring to. To make comparisons they need to have age-standardized rates and ideally, they would compare their data to other LMIC populations.

**Response: We agree and thank the reviewer for raising this. In line 361-368 we have rewritten the recommendation section for clarity as follows: “**Our findings have important implications for health systems in low- and middle-income countries facing rising burdens of chronic multimorbidity. Age-responsive models of care are needed to address the clustering of conditions in older adults. Gender-sensitive interventions should prioritize women’s health across the life course, while providing psychosocial support for individuals experiencing marital disruption. Community sensitization programs aimed at increasing awareness of chronic disease prevention and early detection could further strengthen these efforts. Finally, integrating alcohol-use screening and interventions into primary care may offer a feasible strategy for reducing the burden of multimorbidity.**”**

33. They also make an incorrect claim about “higher levels of education,” which is not backed by the results they presented.

**Response: We thank the reviewer for the comments and have corrected this in the discussion section line 329- 331 as follows “In contrast with studies in some settings (15,30), we found no consistent associations between multimorbidity and income or educational attainment.”**

34. The data availability statement seems inadequate since it makes a vague promise to post the data at some future date. A more precise plan should be shared.

**Response: We have provided the data that reproduces the main results as a supplementary file.**

Reviewer 3

1. Mention the measures Kenya is taking with the recent allocation of the emergency and chronic disease fund scheme under the Social Health Authority and cite accordingly in the introduction.

**Response:. We appreciate this valuable suggestion. However, as our study did not adopt a health system–strengthening perspective and was focused on introducing the concept of multimorbidity rather than individual chronic diseases, we include information on the Social Health Authority’s emergency with regard to Multimorbidity. While the Social Health Authority in Kenya acknowledges the burden of chronic illnesses, there are currently no clear guidelines or policies specifically addressing the management of multimorbidity. Line 71-75 “**Although Kenya’s Social Health Insurance Fund (SHIF) acknowledges the burden of chronic illnesses through initiatives such as the emergency and chronic disease fund, it continues to face implementation challenges, and there are no clear national guidelines or frameworks addressing multimorbidity as a distinct health challenge.**”**

2. While the authors mention paucity of data on the topic, there is actually some work done in Kenya on the general topic of substance use. Consider adding local Kenyan studies to set a good introduction and local data.

**Response: We agree with the reviewer that substance use has been extensively studied in Kenya, and we have included relevant local data from general population studies. However, we note that there is limited local evidence specifically examining the relationship between substance use and multimorbidity.**

3. Similarly, cancer wasn’t mentioned in the introduction as a leading killer disease in Kenya with regard to chronic disease; therefore, it should be noted at least as a limitation. So is HIV status among such a youthful cohort of study participants.

**Response: Cancer is an important contributor to the chronic disease burden in Kenya. However, our study focused on a selected set of chronic conditions aligned with the study objectives. As noted in the limitations section, we acknowledge that not including cancer and other chronic conditions restricts the scope of our findings and does not capture the full spectrum of multimorbidity in the population. Line 348-353 “**This study has important limitations. The inclusion of only a small number of chronic conditions likely led to conservative estimates of multimorbidity prevalence and associations, thus limiting the ability to compare prevalences and associations across studies. This limitation reflects a broader challenge in multimorbidity research, which is marked by methodological variability in population selection, sample representativeness, the range of conditions analyzed, and methods of ascertainment.**”**
